# From fragments to follow-ups: rapid hit expansion by making use of EU-OPENSCREEN resources

**DOI:** 10.1039/d5md00684h

**Published:** 2025-10-22

**Authors:** Laila S. Benz, Jan Wollenhaupt, Aigars Jirgensons, Tanja Miletic, Uwe Mueller, Manfred S. Weiss

**Affiliations:** a Macromolecular Crystallography, Helmholtz-Zentrum Berlin Albert-Einstein-Str. 15 12489 Berlin Germany manfred.weiss@helmholtz-berlin.de; b Laboratory of Structural Biochemistry, Institute of Chemistry and Biochemistry, Freie Universität Berlin Takustrasse 6 14195 Berlin Germany; c Latvian Institute of Organic Synthesis Riga LV-1006 Latvia; d EU-OPENSCREEN ERIC Robert-Rössle Straße 10 13125 Berlin Germany

## Abstract

Quite frequently, it is the progression of initial crystallographic fragment screening hits into more potent binders to their target, which constitutes the major bottleneck in many academic compound or drug development projects. While high quality starting points are critical to the success of a drug development project, it is equally important to have accessible pathways for further compound development. Here, we present two crystallographic fragment screening campaigns using a 96 fragment sub-selection of the European Fragment Screening Library (EFSL) provided by EU-OPENSCREEN. The two campaigns against the targets endothiapepsin and the NS2B–NS3 Zika protease, yielded hit rates of 31% and 18%, respectively. Further, we present how within the framework of the EU-OPENSCREEN European Research Infrastructure Consortium (ERIC) fast identification of follow-up compounds can be realized. With just one round of testing related compounds from the European Chemical Biology Library, two follow-up binders for each of the two targets could be identified proving the feasibility of this approach.

## Abstract 2

Definition and validation of EFSL-96, a 96-membered subset of the European Fragment Screening Library. Hits and follow-up binder identification for endothiapepsin and NS2B–NS3 Zika protease.

## Graphical abstract

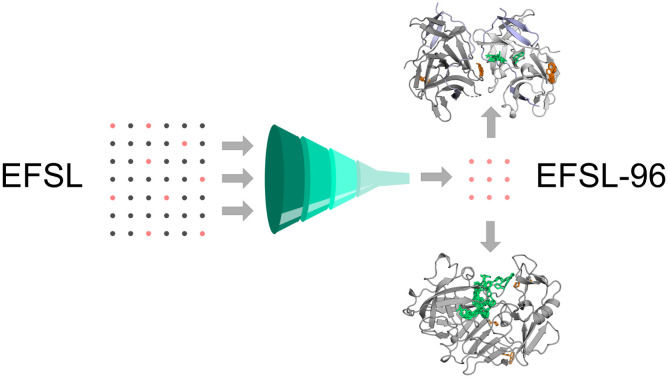

## Introduction

In recent years fragment screening-based approaches have become widely used in drug discovery processes.^1^ Fragments are small molecules loosely following the Astex rule of three.^2^ Due to their small size, they are typically weak binders, but they do bind very efficiently and are easier to progress than larger molecules. Fragment screening campaigns can be conducted, for example using biophysical assays such as microscale thermophoresis (MST), surface plasmon resonance (SPR) or thermal shift assays (TSA). Recently, however, X-ray crystallography has been suggested as the primary approach for fragment screening.^3^ Over the last decade several synchrotron facilities have established X-ray crystallography fragment screening platforms.^4–11^ These fragment screening platforms provide equipment and expertise for the researchers to facilitate the process of fragment screening and make it more accessible to the wider academic and industrial scientific community.

An important hurdle on the way to conducting a fragment screening campaign is the availability of a suitable compound library. To lower the barrier, especially for academia to embark on fragment screening campaigns, several of the screening facilities have also assembled compound libraries, which may be utilized.^12–19^ One such library called the European Fragment Screening Library (EFSL) has recently been assembled by the European Research Infrastructure Consortium (ERIC) EU-OPENSCREEN.^20^ This library consists of 1056 compounds, 968 of them following Astex rule of three and additional 88 so-called minifrags.^13^ Its great advantage is that the compounds are substructures of the EU-OPENSCREEN high-throughput screening library, called the European Chemical Biology Library (ECBL), which contains nearly 100 000 compounds.^20,21^ Consequently, hits derived from the EFSL can readily be progressed to larger compounds, which are contained in the ECBL.

In this work, a representative 96-fragment containing sub-selection of the 1056-compound large European Fragment Screening Library (EFSL-96) has been created. The performance of this subset was validated in two fragment screening campaigns using the model system endothiapepsin (EP) and the NS2B–NS3 protease complex of the Zika virus. EP is an aspartic protease with the catalytic dyad formed by Asp35 and Asp219 and has served as a model protein for finding inhibitors for other aspartic proteases like β-secretase, a key target for treating Alzheimer's disease.^22^ Due to its well-established crystallization and soaking protocol, it has served as a validation target for several crystallographic fragment screening libraries.^3,8,15,23–25^ As a second and real-life target, we addressed the NS2B–NS3 Zika protease which is crucial for the viral life cycle of the Zika virus but with its shallow active site challenging to target. So far there is no specific drug developed against the Zika virus although it is associated with severe neurological complications such as Guillain–Barré syndrome and microcephaly in fetuses and newborns.^26^ A recent study by Ni *et al.* 2025 reported a crystallographic fragment screen using a similar NS2B–NS3 protease construct, underscoring the growing interest in this target.^27^

Rapid follow up of fragment hits for both targets was enabled by selecting small parent molecules from the ECBL, in consultation with EU-OPENSCREEN medicinal chemistry experts. Thus, within one round of off-the-shelf compounds and additional validation of binding poses by crystallography, we achieved two follow-up hits for EP and two follow-ups for the NS2B–NS3 Zika protease, within a very short time and without the need of elaborate computational or synthetic chemistry.

## Material and methods

### Sub-selection and clustering of EFSL

For the sub-selection of the EFSL, all fragments, which are not solid at ambient conditions, were excluded to ensure that they will withstand the drying procedure established in our F2X-facility (see below). All mini-frags were excluded as well. The remaining 734 fragments were further filtered by applying criteria that were also used during the assembly of the F2X-library.^15^ To arrive at a practically manageable set of 96 fragments, compatible with 96-well plate crystallography formats and in line with previous fragment screening studies,^15,25^ the final 546 fragments were submitted to a first MACCS fingerprint-based clustering^28^ with a Tanimoto distance threshold^29^ of 0.635. After this clustering, six small clusters and two singletons were excluded by medicinal chemistry expert inspection. The criteria were not algorithmic but based on common heuristics, such as removal of borderline fragments (according to the rule-of-3 parameters), compounds with unusually high sp^3^-character, low predicted solubility, or unfavorable polar atom distributions. A second round of the clustering of the remaining 531 fragment was then performed, applying a Tanimoto threshold of 0.647. This resulted in 96 clusters. Cluster representatives with the lowest average intra-cluster Tanimoto distance and highest predicted solubility were then selected. One cluster was excluded manually again and 5 more fragments were selected manually out of larger/dissimilar clusters to result in the final 96 fragments. Although more sophisticated approaches (*e.g.* 3D similarity, pharmacophore diversity) exist, the parent library had already been curated for physicochemical and pharmacophoric diversity. Therefore, the additional benefit of advanced clustering was considered limited, and the chosen method provided a fast and reproducible way to maximize structural spread and minimize selection bias. This sub-selection of the EFSL is named ESFL-96.

### Preparation of soaking plates

The library was prepared as dried-on compounds on an MRC 96-well 3-lens low profile plate following the same protocol as previously described for the F2X-universal library.^15^ In short, compounds were provided as 100 mM DMSO-*d*_6_ stocks from the EU-OPENSCREEN ERIC Central Compound Management Facility (CCMF). The compounds were spotted using an ATS Gen 5 Acoustic Transfer System and dried overnight at 42 °C. The fragments in the paper are named according to their name in the European Chemical Biology Database (EDBD).^30^

### Protein purification

#### EP

EP was purified from Suparen® 600 (kindly provided by DSM Food Specialties, Heerlen, The Netherlands) as described previously.^31^ The buffer of the Suparen® 600 material was exchanged to a buffer with 0.1 M sodium acetate pH 4.6. Afterwards the material was further purified by size exclusion chromatography with the same buffer. After purification EP was concentrated to 5 mg ml^−1^, flash frozen in liquid nitrogen and stored at −80 °C until use.

#### NS2B–NS3 Zika protease

The NS2B–NS3 Zika protease construct was cloned into a pET-DUET-1 vector, with residues 46–96 from Zika gene NS2B and the protease site from NS3 (residues 1–177) (courtesy of Martin Scanlon Lab, Monash University) following the design of Li *et al.* 2018, with an engineered C143S mutant to prevent crystallographic disulfide bond formation between neighboring NS3 copies as seen by Lei *et al.*, 2016.^32,33^ The NS3 protease part has an N-terminal His6-tag followed by a TEV protease cleavage site. The plasmid was transformed into BL21(DE3) cells. The transformed cells were used to inoculate an overnight LB culture and then transferred into autoinduction media.^34^ The cells were grown until an OD of 0.8, then cooled to 18 °C and cultured overnight. Cells were harvested by centrifugation and resuspended in buffer A containing 20 mM HEPES, pH 7.8, 250 mM NaCl, 1 mM DTT, 10 mM imidazole, 5% (v/v) glycerol. The cells were then lysed by sonification. The lysate was loaded onto a HisTrap column and after washing with buffer A the protein was eluted with buffer B containing 20 mM HEPES, pH 7.8, 250 mM NaCl, 1 mM DTT, 500 mM Imidazole, 5% (v/v) glycerol. Fractions containing the protein were pooled and dialyzed overnight into 20 mM HEPES pH 7.8, 250 mM NaCl, 1 mM DTT, 5% (v/v) glycerol together with 1 : 20 (w/w) TEV protease to remove the His-tag. After cleavage the protein was further purified with a Ni-NTA column to remove the TEV protease and the uncut protein. The tag-cleaved protein was pooled, concentrated and further purified *via* a size exclusion column into 20 mM HEPES, pH 7.8, 250 mM NaCl, 1 mM DTT, 5% (v/v) glycerol. The protein was then concentrated to 40 mg ml^−1^, flash frozen in liquid nitrogen and stored at −80 °C until use.

### Crystallization

#### EP

EP was crystallized as described previously.^15^ Crystals were grown in sitting drops by mixing 1.5 μl of protein solution (5 mg ml^−1^ in 0.1 M sodium acetate, pH 4.6) with 1.5 μl of the reservoir solution (29% (w/v) PEG 4000, 0.1 M ammonium acetate and 0.1 M sodium acetate, pH 4.6) and 0.2 μl of freshly prepared seed stocks in a dilution of 1 : 15 or 1 : 45. Crystals grew in a few days at 20 °C.

#### NS2B–NS3 Zika protease

The NS2B–NS3 Zika protease was crystallized with some optimizations as described by Huber *et al.*, 2022 by mixing 1 μl protein solution (40 mg ml^−1^ protein in 20 mM HEPES, pH 7.8, 250 mM NaCl, 1 mM DTT, 5% glycerol) with 1 μl reservoir solution (24% w/v PEG 2000, 0.2 M ammonium sulphate and 0.1 M sodium acetate pH 4.6) in hanging drop trays.^35^ Crystals grew in a few days at 20 °C.

### Crystal soaking

#### EP

Soaking experiments were conducted as described previously.^15,36^ In short, 20 μl of soaking solution containing 19.8% (w/v) PEG-4000, 68 mM ammonium acetate, 19.3% (v/v) glycerol and 9% (v/v) DMSO were added to the fragment plate. Fragments were resolubilized in the soaking solution and for each fragment one crystal was incubated overnight. No additional cryo-protection step was necessary as the soaking solution is already cryo-protectant.

#### NS2B–NS3 Zika protease

For soaking of the NS2B–NS3 Zika protease crystals 40 μl of soaking solution containing 0.1 M sodium acetate pH 4.6, 0.2 M ammonium sulfate, 24% (w/v) PEG 2000 and 5% (v/v) DMSO was added to the reservoir. 0.4 μl of soaking buffer was added onto each dried fragment and incubated overnight with one crystal. 40 crystals were soaked only in the soaking buffer to generate apo datasets. Before fishing NS2B–NS3 Zika protease crystals were cryo-protected by incubation in a solution containing 0.1 M sodium acetate pH 4.6, 0.2 M ammonium sulfate and 30% (w/v) PEG 2000.

### Data acquisition and processing, refinement and hit identification

#### EP

Diffraction datasets were acquired on beamline BL14.1 at BESSY II in Berlin.^37^ For each crystal, a 180° rotation using an incremental step of 0.2° and an exposure time of 0.05 s with an aperture of 100 μm was measured. All datasets were auto processed within FragMAXapp^38^ using XDSAPP.^39^ For structure solution the datasets were afterwards auto refined with the fspipeline script.^40^ As a starting model for auto refinement the PDB entry 4Y5L^41^ was used. The automatically refined datasets were analyzed with PanDDA^42^ to identify fragment hits. The fragment hits were modelled by inspection of the respective dataset in COOT.^43^

For the EP follow-up compounds, datasets were collected on beamline BL14.2 at BESSY II in Berlin.^37^ All crystals were measured with a 240° rotation using an incremental step of 0.1° and an exposure time of 0.05 s. Datasets were processed, refined and analysed as for the fragment screen datasets described above.

#### NS2B–NS3 Zika protease

Diffraction data was collected on beamline 14.1 at BESSY II in Berlin. For each crystal a 160° rotation using an incremental step of 0.2°, an exposure time of 0.1 s and an aperture of 100 μm was measured. As for EP the datasets were analysed within the FragMAXapp. Datasets were auto processed with XDSAPP and auto refined using the fspipeline script and DIMPLE.^44^ As input model an in-house refined dataset was used based on PDB entry 7ZUM.^45^ PanDDA and LigandFit^46^ were used for hit identification. As for EP the fragment hits were modelled by inspection of the respective dataset in COOT.

For the NS2B–NS3 Zika protease the follow-up compounds were measured and analysed the same way as for the initial fragment screen.

### Selection of follow-up compounds

Each of the fragment hits selected for follow-up search was subjected to a substructure search in the ECBL (98 560 compounds) hosted by the ECBD (https://ecbd.eu/). If the number of hit analogues exceeded 200, the search was limited to compounds bearing no more than 4 H-bond donor and 4 H-bond acceptors. The analogues for crystallographic evaluation were further selected manually considering their potential for picking up additional interactions. For this purpose, the identical substructure of each of the analogue and its original fragment were overlaid using PyMol for visualization of a fragment–enzyme complex. The analogues showing obvious steric clashes with the enzyme or pointing out of the active site cavity of the enzyme were filtered out. The remaining analogues were chosen for validation by X-ray crystallography.

### Thermal shift assay

#### EP

Thermal shift assay measurements (TSA) with initial fragment hits and their follow-up binders were carried out in triplicate in the CFX96 Real-Time System (Bio-Rad). Identified follow-up compounds and their initial fragments were measured at 5×, 10× and 50× molar excess relative to EP. Samples contained 5 μM EP in buffer (0.1 M sodium acetate pH 4.6, 0.01% (v/v) Tween20), 0.2% (v/v) SYPRO Orange (5000× stock in DMSO, Invitrogen) and 25 μM, 50 μM or 250 μM fragment or follow-up compound in DMSO (1% v/v) in a volume of 25 μl. As control 1% (v/v) DMSO was used. Samples were heated from 4 °C to 95 °C in 0.1 °C increments. Melting temperatures were determined by analysis of the first derivative of the fluorescence/temperature function.

#### NS2B–NS3 Zika protease

TSA measurements and analysis were conducted as described for EP, with some modifications. NS2B–NS3 Zika protease was measured at a concentration of 15 μM, in a buffer containing 20 mM HEPES pH 7.3, 200 mM NaCl and 1 mM DTT with 0.4% (v/v) SYPRO Orange (5000× stock in DMSO, Invitrogen). Tested initial fragments and corresponding follow-up binders were measured at a concentration of 75 μM, 150 μM and 750 μM in DMSO (1% v/v/).

### Final refinement and data deposition

For both campaigns the structures containing the hits were subjected to one round of manual refinement including the modelled ligand using phenix.refine.^47^ The structures along with the experimental structure factor amplitudes were deposited in the PDB as group depositions with the following IDs:

G_1002349 (EP)

G_1002347 (NS2B–NS3 Zika protease)

Follow-up hits are deposited as single depositions to the PDB with the following PDB codes:

9RFX, 9RG6 (EP), 9RGA, 9RGF, 9RGG (NS2B–NS3 Zika protease)

For the EP hits, which could only be detected using PanDDA event maps, the respective PanDDA event- and Z-maps are included in their respective PDB entry. Fragment screening data will also be linked to the ECBD.

## Results

### Selection of a representative 96-compound subset (ESFL-96) of the ESFL

**Fig. 1:**
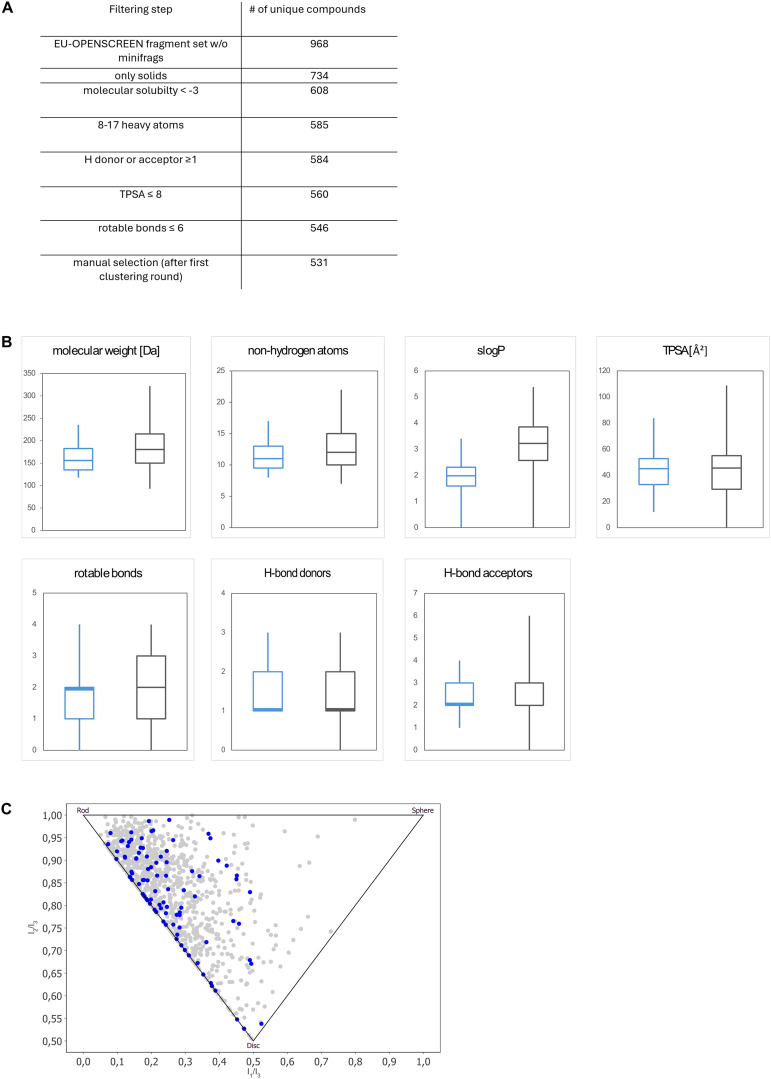
The newly introduced ESFL-96 library is a 96-compounds large subset of the ESFL. A Filtering steps B molecular properties of the library shown as boxplots (ESFL-96 blue, ESFL, grey) C PMI plot comparing the ESFL-96 library (blue) with the complete ESFL (grey).

To assemble a representative 96-compound sub selection of the 1056 compound-membered ESFL, criteria similar to the F2X-Universal library^15^ as well as a clustering approach were used. The initial filtering steps conducted are shown in Fig. 1A. An overview of molecular properties usually used to characterize compound libraries is given in Fig. 1B. The 96 selected fragments represent the chemical diversity of the initial fragment library as shown by the PMI plot in Fig. 1C. The compounds constituting the ESFL-96 are available in Table S1.

### Screening hits obtained against EP

**Fig. 2:**
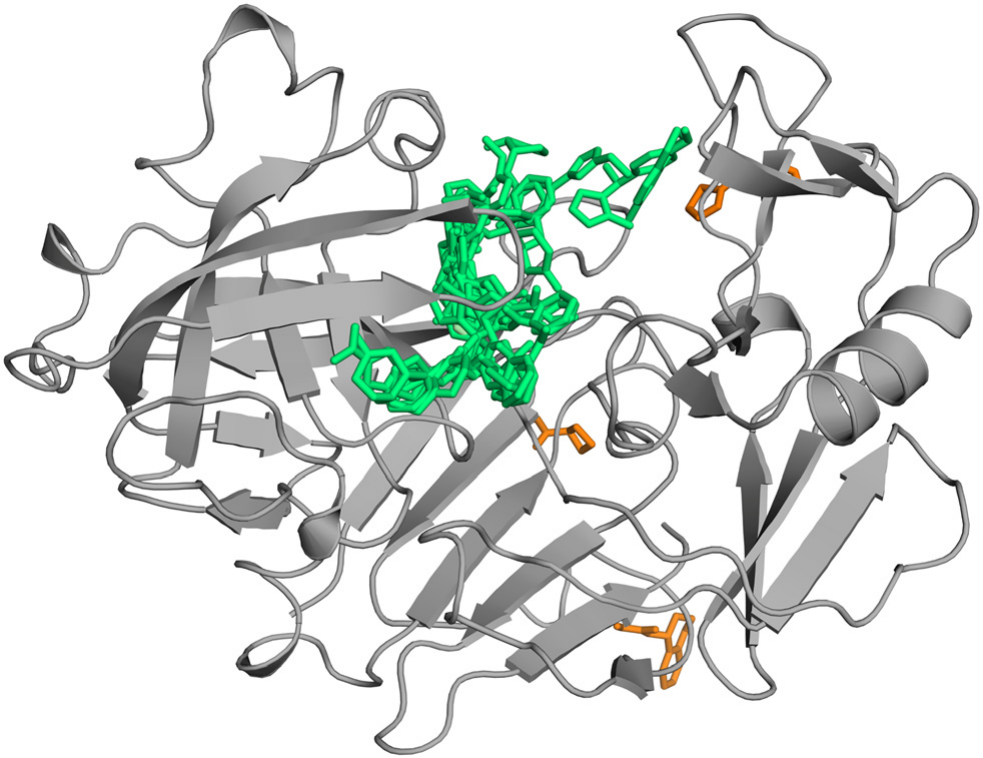
The crystallographic compound screen against EP yielded 30 hits. Overview of all hits, cleft binders shown in green and remote binders in orange.

EP is an aspartic protease with the catalytic dyad formed by Asp35 and Asp219. In our fragment screen against the ESFL-96 selection we identified 30 binding fragments, with a total of 42 binding events (Fig. 2). The fragments are well distributed over the entire peptide recognition cleft. All but three binding events are within the peptide recognition cleft. The remaining three of the fragment binding events occupy additional remote binding sites.

**Fig. 3:**
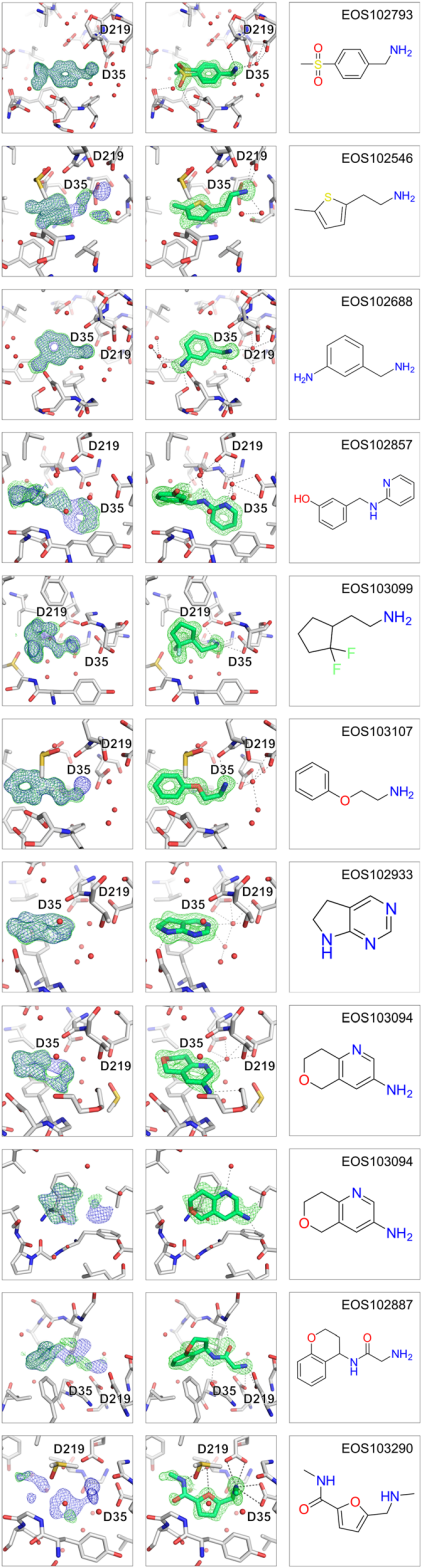
The selected fragment hits for follow-up search are depicted. The three panels from left to right show first the (2mF_o_-DF_c_, *α*_c_) maps contoured at *σ* = 1 (blue) and the (mF_o_-DF_c_, *α*_c_) maps contoured at *σ* = 3 (green) after auto-refinement with fspipeline, the second panel the omit-map around the ligand after refinement with the ligand placed and third the chemical structure of the fragment hit. The catalytic residues Asp35 and Asp219 are indicated.

Eleven of the fragments interact via H-bonds with the catalytic dyad and additional eleven hits form H-bonds with the catalytic water, interacting indirectly with the catalytic dyad. Most fragments bind to the S1 pocket (21 binding events) or bind over two pockets and bind to the S1 and S2 or S3 pocket (6 more). The S2′ pocket is occupied by 5 fragments and the remaining 8 fragments are distributed over the S1′, S2′ and S3 to S6 binding pocket. For selection of follow-up compounds out of the ECBL ten direct and water-mediated catalytic dyad binders were selected based on their clear binding evidence. The selected binders EOS102793, EOS102546, EOS102688, EOS102857, EOS103099, EOS103107, EOS102933, EOS103094, EOS102887 and EOS103290 are shown in Fig. 3, all other fragment hits are depicted in Fig. S1. For rapid expansion of the hits potential follow-up compounds were found in the ECBL by substructure search of the hits in the diversity library available in the ECBD and a subsequent manual selection resulting in 3–6 suggested compounds for crystallography studies for each 5 out of the 10 selected hits. The compounds were screened with X-ray crystallography for follow up hit identification. For the selection process a limit of maximum four H-bond donors and four H-bond acceptors was set, and the found compounds were manually filtered for interesting expansion vectors based on the structural data of the fragment hits.

### Two stronger EP binders could be identified

**Fig. 4:**
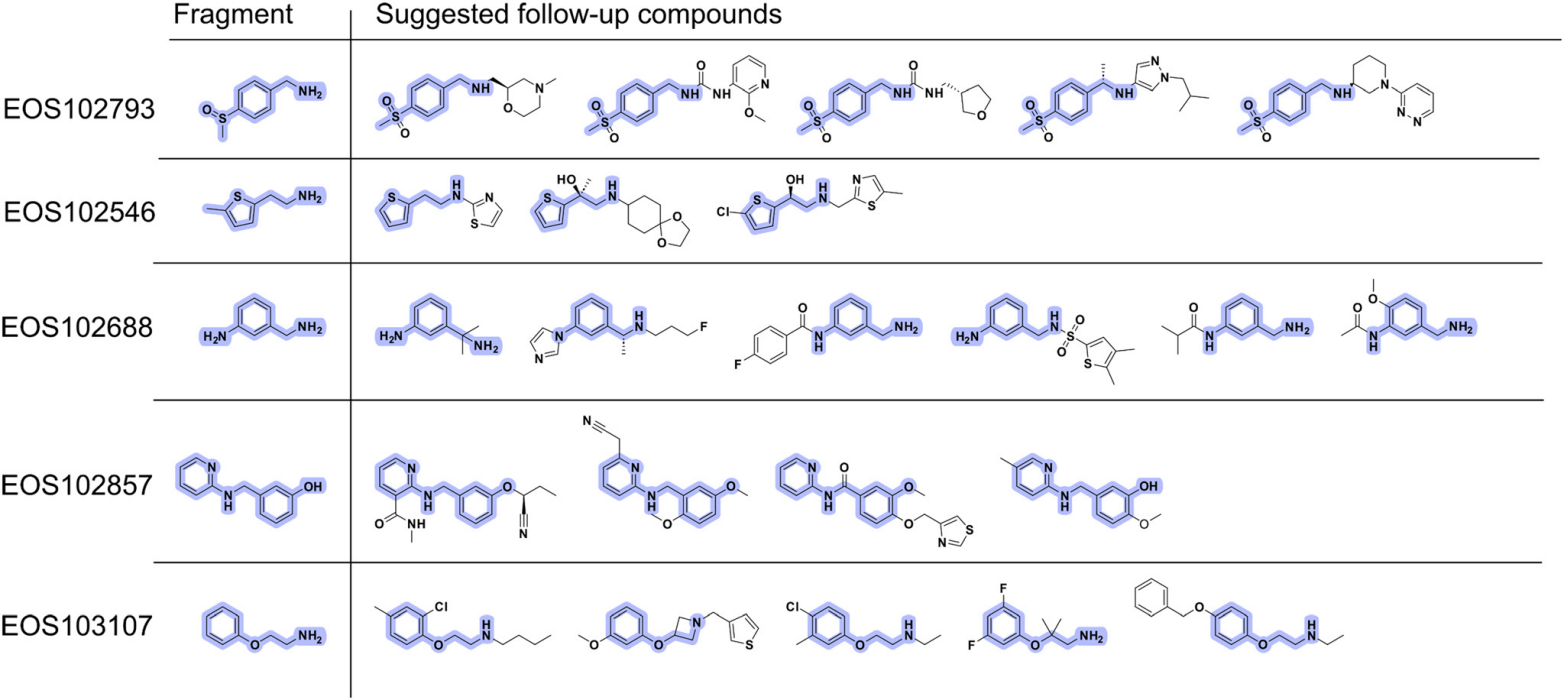
Overview of 2D structures of proposed EP follow-up compounds from the ECBL library. The maximum common substructure (MCS) between starting fragment and respective follow-ups is highlighted in violet.

The potential follow-up compounds are shown in Fig. 4, highlighting the chemical diversity of the proposed follow-up compounds. Out of the 23 proposed follow-up compounds six could be identified as crystallographic binders.

**Fig. 5:**
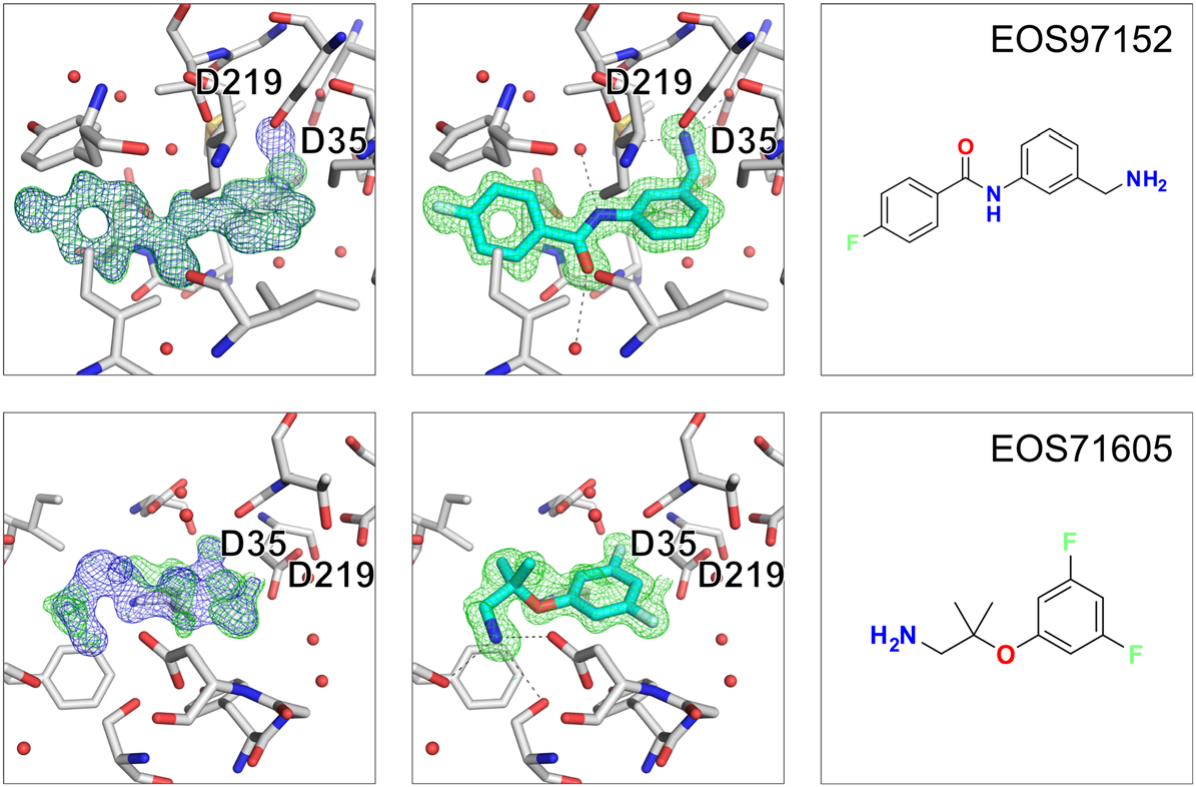
Two follow-up compounds were found by X-ray crystallography. Close-up of crystallographic follow-up hits. The three panels from left to right show first the (2mF_o_-DF_c_, *α*_c_) maps contoured at *σ* = 1 (blue) and the (mF_o_-DF_c_, *α*_c_) maps contoured at *σ* = 3 (green) after auto-refinement with fspipeline, the second panel the omit-map around the ligand after refinement with the ligand placed in green and last the chemical structure of the fragment hit. The catalytic residues Asp35 and Asp219 are annotated.

Five out of the twenty-three proposed follow-up compounds bind in the peptide cleft of EP. The five follow-up compounds are based on three fragment hits, EOS102793, EOS102688 and EOS103107. The five binders were identified by analyzing the data with PanDDA and manual inspection of the datasets. Follow-up compounds EOS97152 and EOS71605 are clearly binding to the protein and are binding with full occupancy whereas the remaining follow-up compounds are binding with lower occupancy than the initial fragments. The electron density maps, and the binding mode of the follow-up compounds EOS97152 and EOS71605 are shown in Fig. 5, the other three hits (EOS44278, EOS49905 and EOS12514) are depicted in Fig. S2.

Follow up hit EOS97152 is based on fragment EOS102688. The hit compound EOS97152 replaces the catalytic water with a primary amine now directly interacting with the catalytic dyad. Compared to the initial fragment hit the binding pose is quite substantially shifted, the new compound extends from the S3′ towards the S2 pocket of EP whereas the initial binding fragment is in the S1 pocket.

**Fig. 6:**
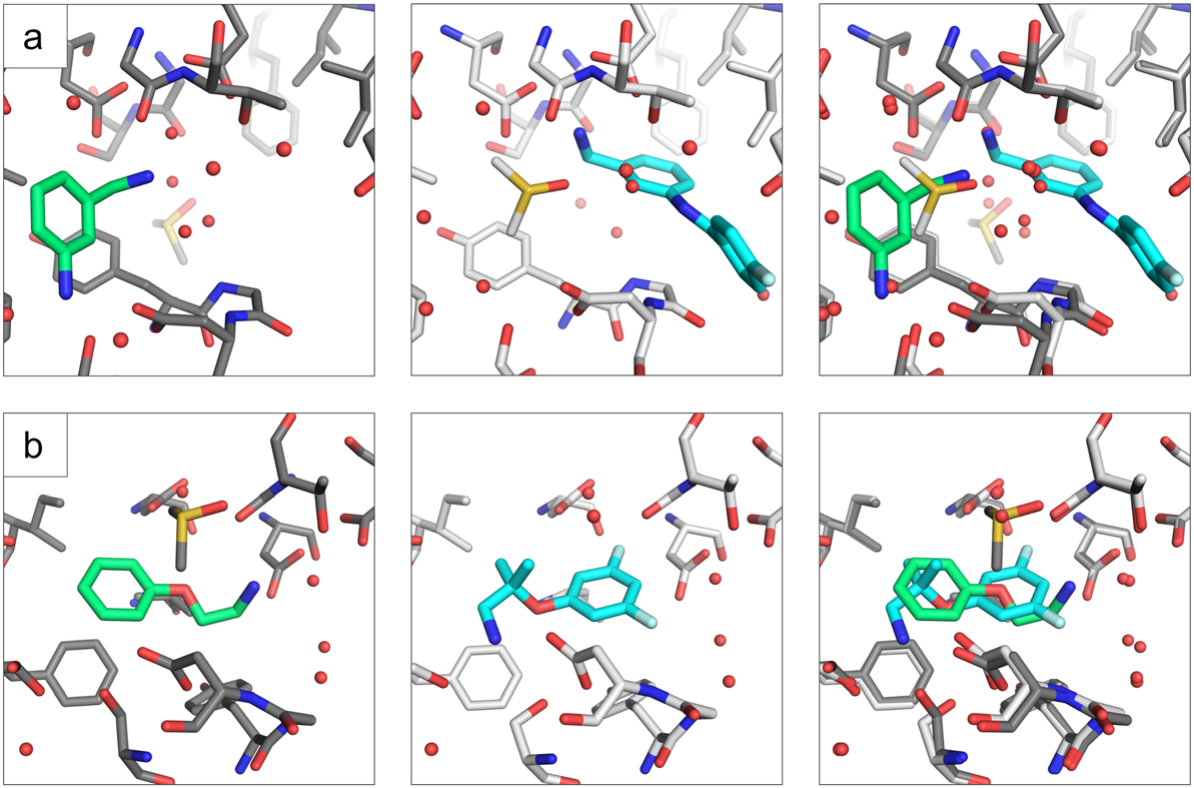
Comparison of initial fragment hit binding pose and follow-up compound binding pose. The three panels from left to right show first in green the initial fragment hit, in the middle the follow-up compound in cyan and in the last panel an overlay of fragment hit and follow-up compound. Shown are from top to bottom EOS97152 (a), and EOS71605 (b).

The follow-up compound EOS71605 based on fragment hit EOS103107 binds in the same sub pocket as the initial fragment hit did. Compared to the initial fragment hit, EOS71605 binds in 180° flipped way to EP, thereby it picks up new H-bonds with Ser115, Ser83 and Asp81 but at the same time it does not longer interact with the catalytic water, resulting in a rather high RMSD of 4.8 Å for the maximum common substructure (MCS). All mentioned interactions are shown in Fig. 5 and a comparison of the binding poses between initial fragment hit and follow up hit is depicted in Fig. 6, for the additional low occupancy binder interactions and binding mode are depicted in Fig. S2 and S3. The compounds EOS97152 and EOS71605 along with their initial fragments, were assessed by TSA, but no temperature shift was observed.

### Screening hits obtained against NS2B–NS3 Zika protease

**Fig. 7:**
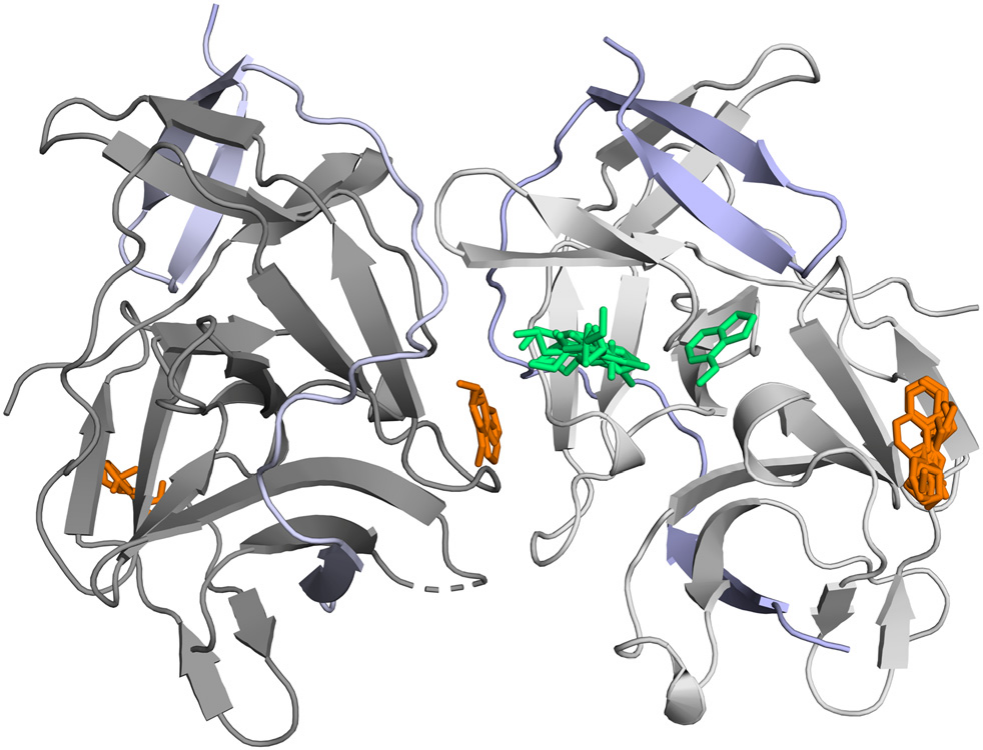
The crystallographic compound screen against the NS2B–NS3 Zika protease yielded 18 hits. Binders at or close to the active site are shown in green, remote binders in orange. The NS2B co-factor is colored in chain A and C in light blue, the NS3 protease domain is colored in dark grey in chain B and in light grey in chain D.

Our second validation target for the ESFL-96 is the NS2B–NS3 Zika protease. The protein construct contains the soluble part of the membrane bound NS2B co-factor and the protease domain of the NS3 protease/helicase protein of the Zika virus. The NS2B–NS3 Zika protease is a heterodimer, the NS2B co-factor wraps around the NS3 protease domain and forms part of S2 and S3 pocket.^48^ The NS2B–NS3 Zika protease crystallizes in our crystallization condition with two molecules in the asymmetric unit, the NS2B-cofactor is assigned chain IDs A and C, the NS3 protease domain chain IDs B and D. The N-terminus of the second NS3 protease domain (chain D) binds to the active site of the molecule with chain IDs A and B in the asymmetric unit as reported by Zhang *et al.* 2016.^48^ It is a serine protease, and the catalytic triad is formed by Ser135, His51 and Asp75. For the second fragment screening campaign LigandFit and PanDDA were used to identify binding events. With this campaign 18 fragments were identified as crystallographic binders with a total of 22 binding events. Out of the 22 binding events 10 fragments are binding in the S1 subpocket of the active site close to the catalytic triad forming π–π stacking interaction with Tyr161. One of the fragments interacts directly with the catalytic His51. Eleven of the binding events are at crystal–contact interface sites. All hits are shown in Fig. 7 with the fragment hits close or at the active site in green and the remote binders in orange.

**Fig. 8:**
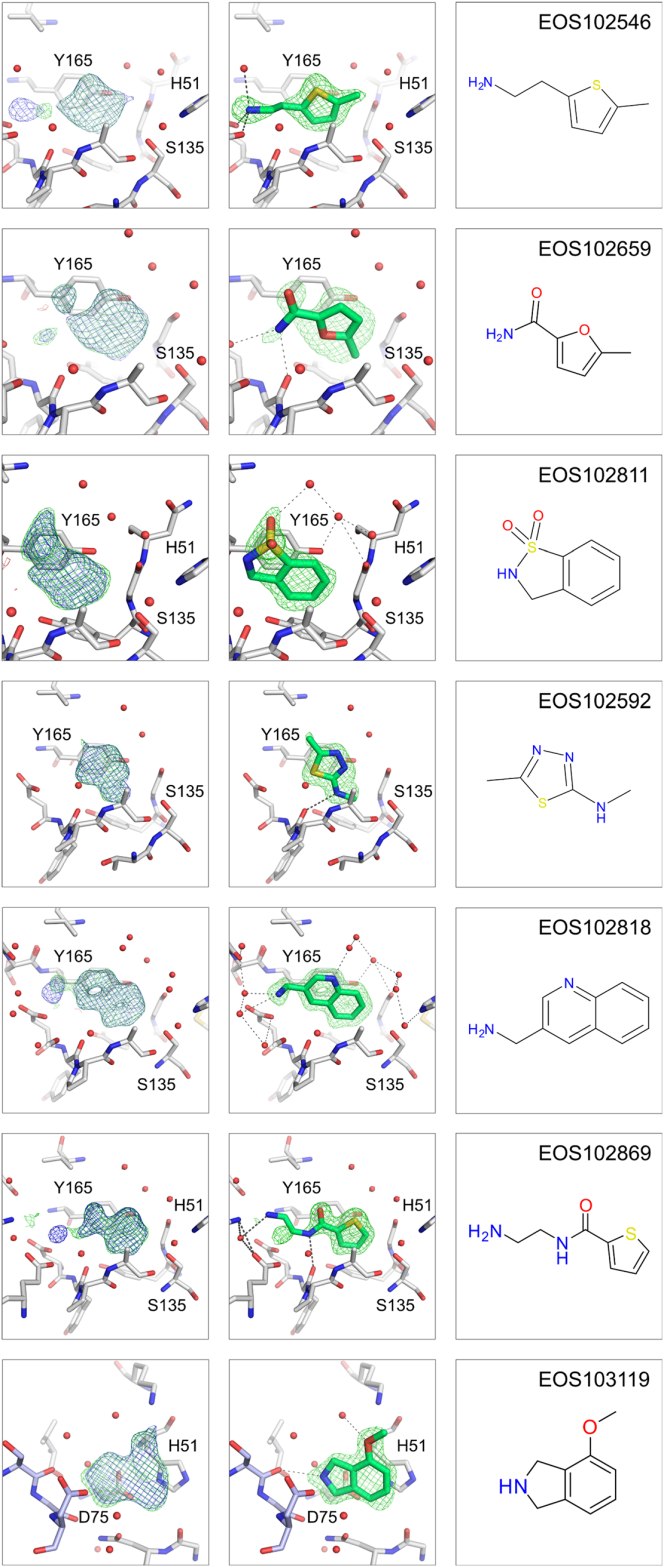
The selected fragment hits for follow-up search are depicted. The three panels from left to right show first the (2mF_o_-DF_c_, *α*_c_) maps contoured at *σ* = 1 (blue) and the (mF_o_-DF_c_, *α*_c_) maps contoured at *σ* = 3 (green) after auto-refinement with fspipeline, the second panel the omit-map around the ligand with the ligand placed and third the chemical structure of the fragment hit. The catalytic residues His51, Asp75 and Ser135 as well as Tyr165 all from the NS3 protease are annotated. The NS3 protease part is shown in grey and the NS2B co-factor in light blue.

For expansion of the fragment hits seven representative hits close by or at the active site were selected. The seven chosen fragment hits are shown in Fig. 8, all other fragment hits are shown in Fig. S3. Fragments EOS102546, EOS102659, EOS102811, EOS102592, EOS102818 and EOS102869 form π–π stacking interaction with Tyr161. Fragment EOS103119 interacts *via* π–π stacking with the catalytic His51. For rapid expansion of the hits potential follow-up compounds were found in the ECBL by the substructure search of compounds available in the ECBD and a subsequent manual selection resulting in 2–21 suggested compounds for crystallography studies for each hit. For the selection process a limit of maximum four H-bond donors and four H-bond acceptors was set, and the found compounds were manually filtered for interesting expansion vectors based on the structural data of the fragment hits. The compounds were screened with X-ray crystallography for follow up hit identification.

### Follow-up compounds binding NS2B–NS3 Zika protease

**Fig. 9:**
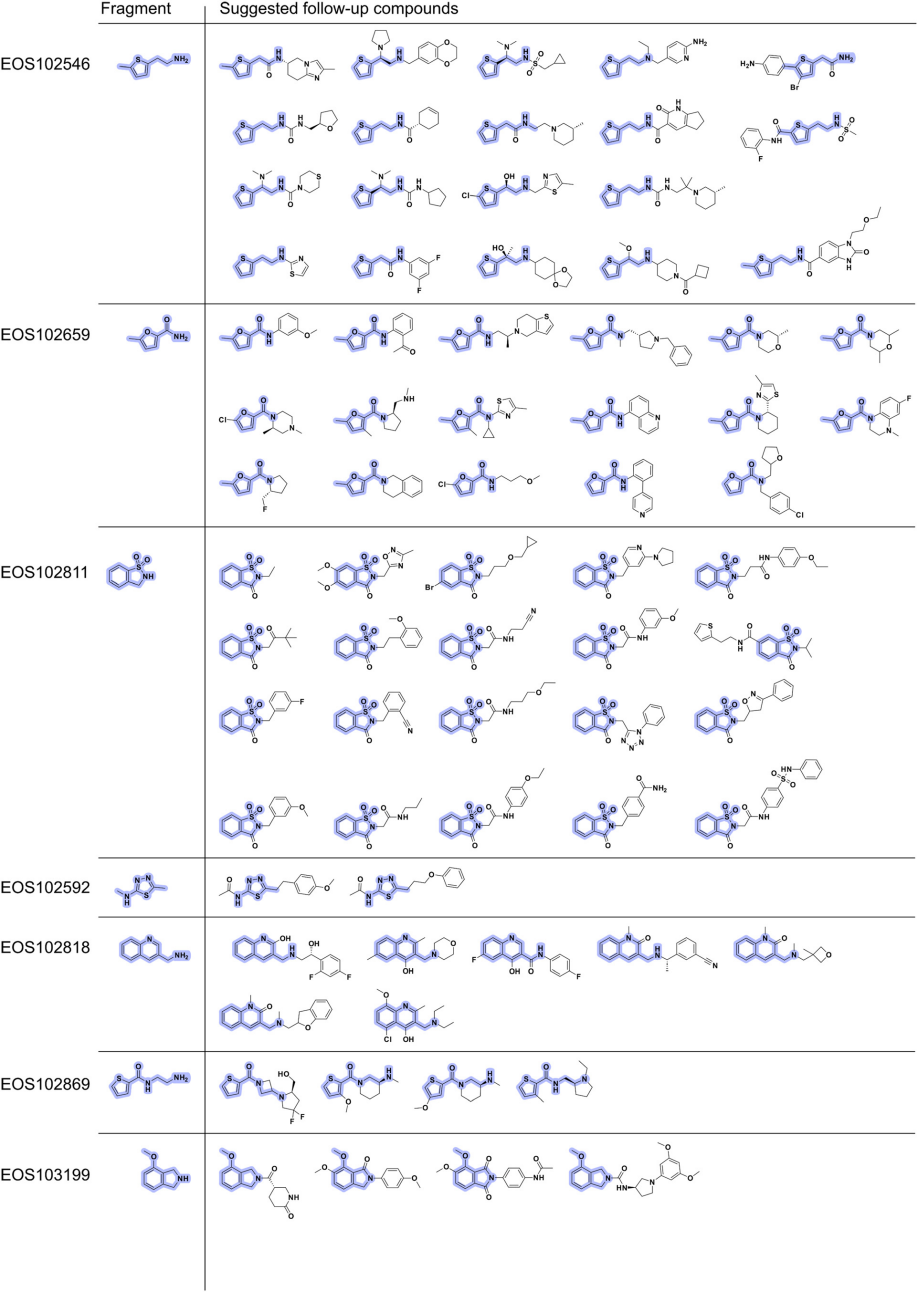
Overview of 2D structures of proposed NS2B–NS3 Zika protease follow-up compounds from the ECBL library. The maximum common substructure (MCS) between starting fragment and respective follow-ups is highlighted in violet.

The 75 potential follow-up compounds selected from the ECBL are shown in Fig. 9. As is evident from the chemical structures, the selected compounds are structurally highly diverse. All selected compounds were screened by X-ray crystallography to identify new binders.

**Fig. 10:**
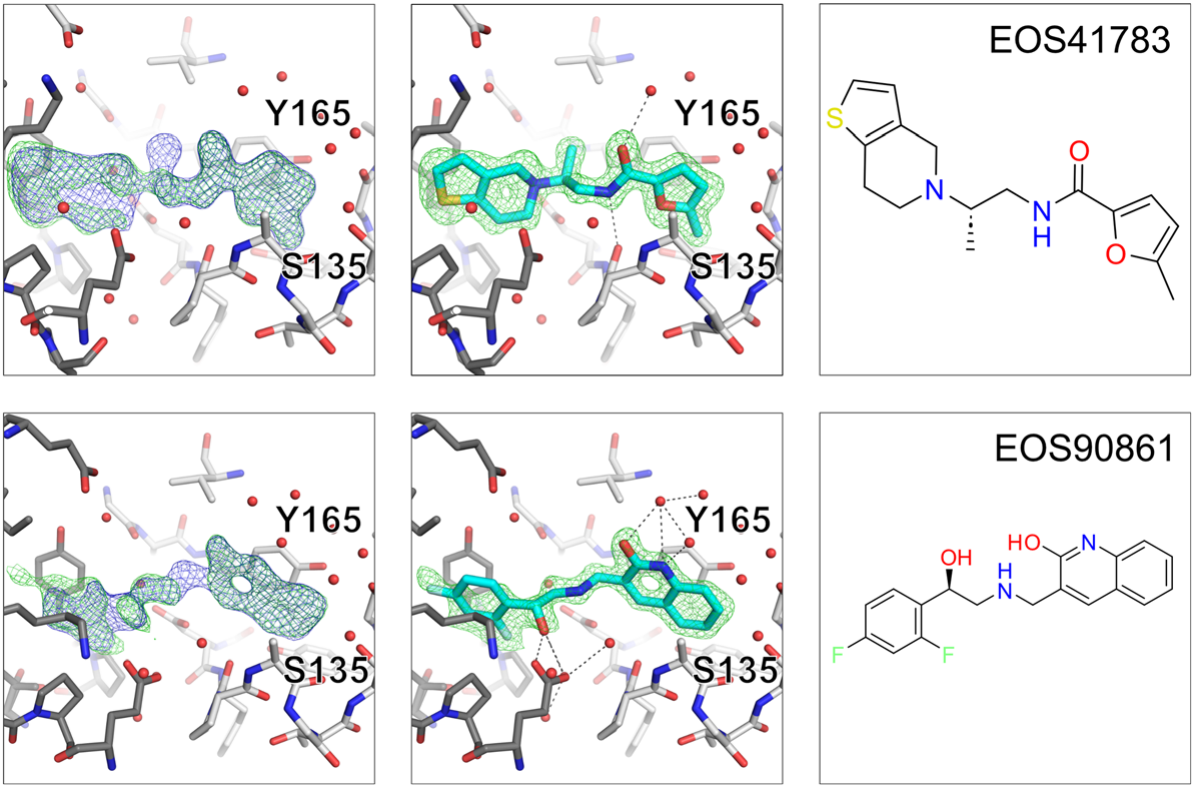
NS2B–NS3 Zika protease follow-up hits with detail presentation of follow-up hits. The three panels show from left to right the electron density map after auto-refinement with fspipeline, the (2mF_o_-DF_c_, *α*_c_) maps are contoured at *σ* = 1 (blue) and the (mF_o_-DF_c_, *α*_c_) maps contoured at *σ* = 3 (green), next the ligand modelled into the omit map after refinement and last the chemical structure of the binder.

Three of the follow-up compounds were identified as crystallographic binders. One of the hits was found at a new binding site at a crystal–crystal contact site (see Fig. S5) and two of them exhibit the initial binding mode of the fragment hit. The newly identified binders are clearly visible in the electron density as shown in Fig. 10, where the two binders are shown with the difference density map after auto-refinement and with the ligand omit map after refinement. Both newly found binders extend towards the second copy of the protein in the asymmetric unit of the crystal lattice.

**Fig. 11:**
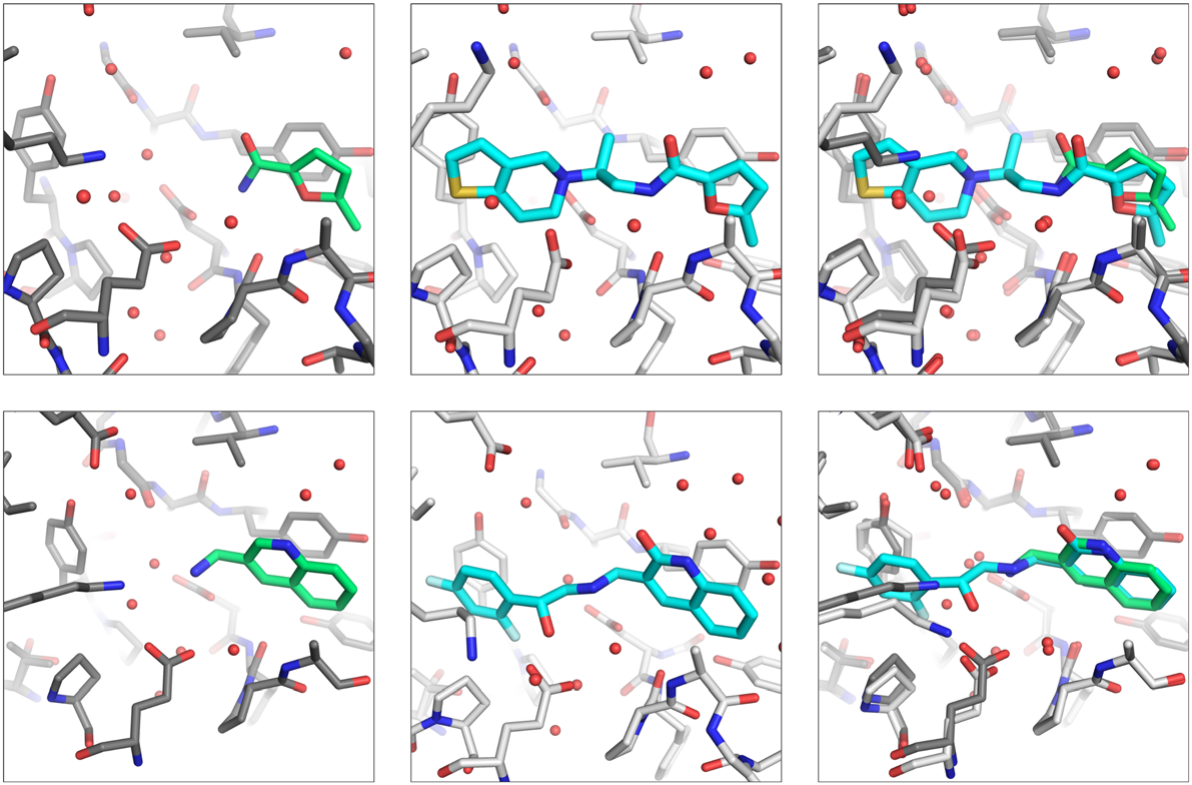
Comparison of initial fragment hit binding pose and follow-up compound binding pose. The three panels from left to right show first in green the initial fragment hit, in the middle the follow-up compound in cyan and in the last panel an overlay of fragment hit and follow-up compound. Overlay follow-up hits with initial fragment hit, first row left EOS102659 (green), mid EOS41783 (cyan) and second row right EOS102818 (green) and mid EOS90861 (cyan).

The binder based on fragment hit EOS102659, EOS41783, is slightly tilted compared to the initial fragment hit with an RMSD of 1 Å between MCS of fragment hit and initial binder. Whereas the second binder, based on fragment hit EOS102818, EOS90861 keeps the initial binding mode exactly with an RMSD of 0.37 Å between the MCS of fragment hit and follow-up binder (Fig. 11). Both follow-up hits form new H-bonds and thereby bind stronger to the protein. Binder EOS41783 forms a new H-bond with the backbone of Tyr130D and interacts *via* π–π stacking with Tyr130B. Compound EOS90861 forms a new H-bond with Asp129D and interacts as well *via* π–π stacking with Tyr130B. The compounds EOS41783 and EOS90861, as well as the fragments on which they are based, were assessed by TSA, but no temperature shift was observed.

## Discussion

Crystallographic fragment screening is developing into a more routine experiment and constitutes an attractive starting point for drug design. Next to the infrastructure needed and a suitable crystal system for the target, the success of crystallographic fragment screening relies heavily on a suitable library.

Our ESFL-96 selection is representative of the larger EU OPENSCREEN fragment library ESFL and has a comparable 3D diversity as other crystallographic fragment screening libraries such as the F2X-Universal library^15^ or the DSIpoised library.^12^ Here we have shown that for both targets, EP and the NS2B–NS3 Zika protease high hit rates of 31% and 19% could be achieved. Our observed hit rate for EP is on the upper range as for other screens with EP (30% against F2X-Entry library,^15^ 10% against the older HZB library,^25^ 20% against the Marburg 361-compound library).^3^ The library contains only 96 representative fragments, making it feasible to screen the whole library in duplicates typically in less than 24 h synchrotron beamtime.

Obtaining high hit rates of diverse fragments is just the first step for successful compound development. In recent years there have been different approaches for facilitating the first steps after fragment hit identification by crystallography. One approach is to screen libraries containing chemical handles for easier follow-up chemistry,^12,49^ or to use parallel-in plate chemistry to screen again a larger library generated by fast parallel in-plate chemistry.^50–52^ A different approach focusing on academic research groups with limited resources is the Frag4Lead workflow which makes use of template based-docking and selection of readily available commercial compounds helping to rapidly increase affinity of fragment hits.^36^

Here, we present the possibility of progressing initial fragment hits to larger binders by making use of a highly diverse small molecule library (ECBL). For the 96 fragments of the ESFL-96 fragment library, on average 311 larger compounds are available, with a total of nearly 30 000 larger compounds being available. All these compounds are readily accessible for European and international scientists through the EU-OPENSCREEN, which provides follow-up consultation with medicinal chemistry groups. Critical medicinal chemistry expertise supports the selection of follow-up compounds, making this framework especially valuable for academic groups lacking in-house medicinal chemistry capabilities.

With this concept we identified two clear binders and three additional larger binders for EP. The hits provide useful information about favorable binding modes and the low affinity binders provide interesting insides in which pharmacophores might be favorably facilitating the next round of directed compound design.

For NS2B–NS3 Zika protease we found a total of three binders, two of them keeping the initial binding mode of the starting fragment. With RMSD values within 1 Å of the fragment core structure and clear binding evidence, the two binders provide valuable information for further compound development. Both binders block the S1 sub pocket of the NS2B–NS3 Zika protease. A limitation of searching for follow-up compounds in an existing library as used here is that not all compounds might have every exit vector covered. To develop a strong NS2B–NS3 Zika protease inhibitor it might be interesting for the next compound expansion steps to focus compound optimization and growing towards the catalytic triad and other sub pockets of the protease. For both campaigns the follow-up compounds provide promising starting points for further optimization, despite the fact that a TSA analysis did not result in any measurable effect. It is quite common, however, that crystallographic fragment hits cannot be orthogonally validated in other biophysical assays. This was demonstrated systematically and convincingly by Schiebel and colleagues.^3^ Next steps would now include rational compound expansion and experimental validation of the binding affinity and inhibitory potential of the compounds.

With our identified follow-up hits we demonstrated that the collaboration with EU-OPENSCREEN through their partners and their resources provides the opportunity for fast hit expansion and thereby helps to tackle the bottleneck of academic drug design which could help to accelerate the development of new tool compounds and drugs.

## Conflicts of interest

There is no conflict of interest to declare.

## Supplementary Material

MD-OLF-D5MD00684H-s001

MD-OLF-D5MD00684H-s002

MD-OLF-D5MD00684H-s003

MD-OLF-D5MD00684H-s004

## Data Availability

The coordinates and structure factors have been deposited in the Protein Data Bank. Both fragment screening campaigns are deposited as group depositions (G_1002349 for EP, G_1002347 for NS2B–NS3 Zika protease), follow-up hits are deposited with the following accession codes: 9RFX, 9RG6 (EP), 9RGA, 9RGF, 9RGG (NS2B–NS3 Zika protease). Other data are available from the corresponding authors upon request. Supplementary information: the supplemental information contains files with the supplemental figures, the ECBL-96 compound list, ECBL EP follow up compound list and the ECBL NS2B–NS3 follow up compound list. All compound lists contain the fragment 2D structures, SMILES codes and ECBL-IDs of all screened compounds. See DOI: https://doi.org/10.1039/d5md00684h.
