# Supplementary material for: From fragments to follow-ups: rapid hit expansion by making use of EU-OPENSCREEN resources
Source: RSC Med Chem. 2025 Oct 22;16(12):6190–203. doi: 10.1039/d5md00684h (PMC12542027; doi:10.1039/d5md00684h)
Supplement: MD-016-D5MD00684H-s001 [file MD-016-D5MD00684H-s001.pdf]

Supplementary Information (SI) for RSC Medicinal Chemistry.
This journal is © The Royal Society of Chemistry 2025

Supplemental figures for Bridging Fragments and Follow-Ups: Crystallographic Screening of the ECBL-96 library to leverage EU-OPENSCREEN Resources

Laila S. Benz1,2), Jan Wollenhaupt1,3), Aigars Jirgensons4), Tanja Miletic5), Uwe Mueller1) & Manfred S. Weiss1,*)

1 Macromolecular Crystallography, Helmholtz-Zentrum Berlin, Albert-Einstein-Str. 15, 12489 Berlin, Germany

2 Freie Universität Berlin, Institute for Chemistry and Biochemistry, Structural Biochemistry Group, Berlin, Germany

3 present address: Proteros Biostructures GmbH, Martinsried, Germany

4Latvian Institute of Organic Synthesis, Riga LV-1006, Latvia

5EU-OPENSCREEN ERIC, Robert-Rössle Straße 10, 13125 Berlin, Germany

* corresponding author

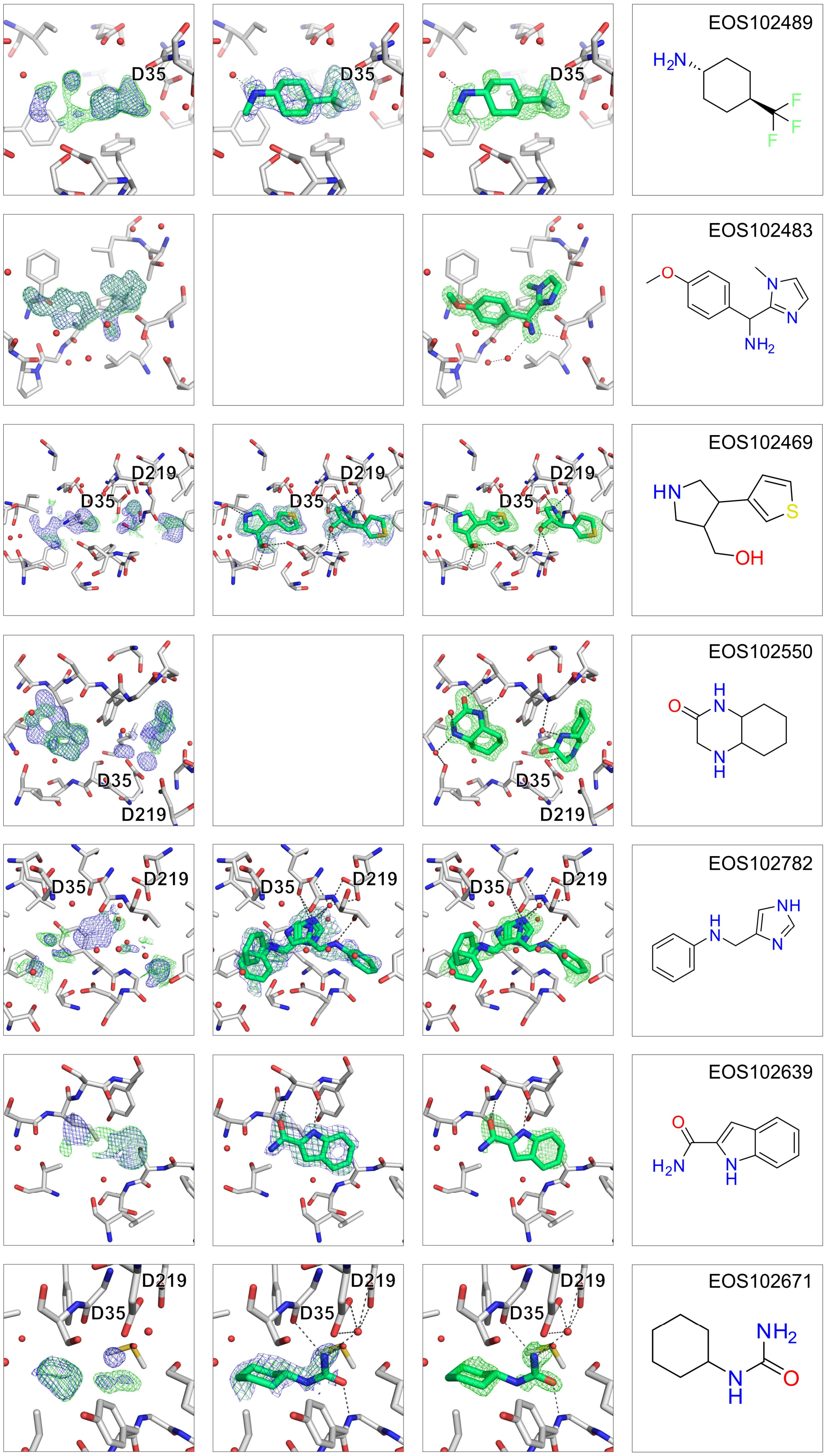
D35
D35
D35
EOS102489
H2N
F
F
F
EOS102483
O
N
N
NH2
D219
D35
D219
D35
D219
D35
EOS102469
HN
S
OH
D35
D219
D35
D219
EOS102550
H
O
N
N
H
D35
D219
D35
D219
D35
D219
EOS102782
H
N
NH
N
EOS102639
O
H2N
N
H
D219
D35
D219
D35
D219
D35
EOS102671
NH2
N
H
O

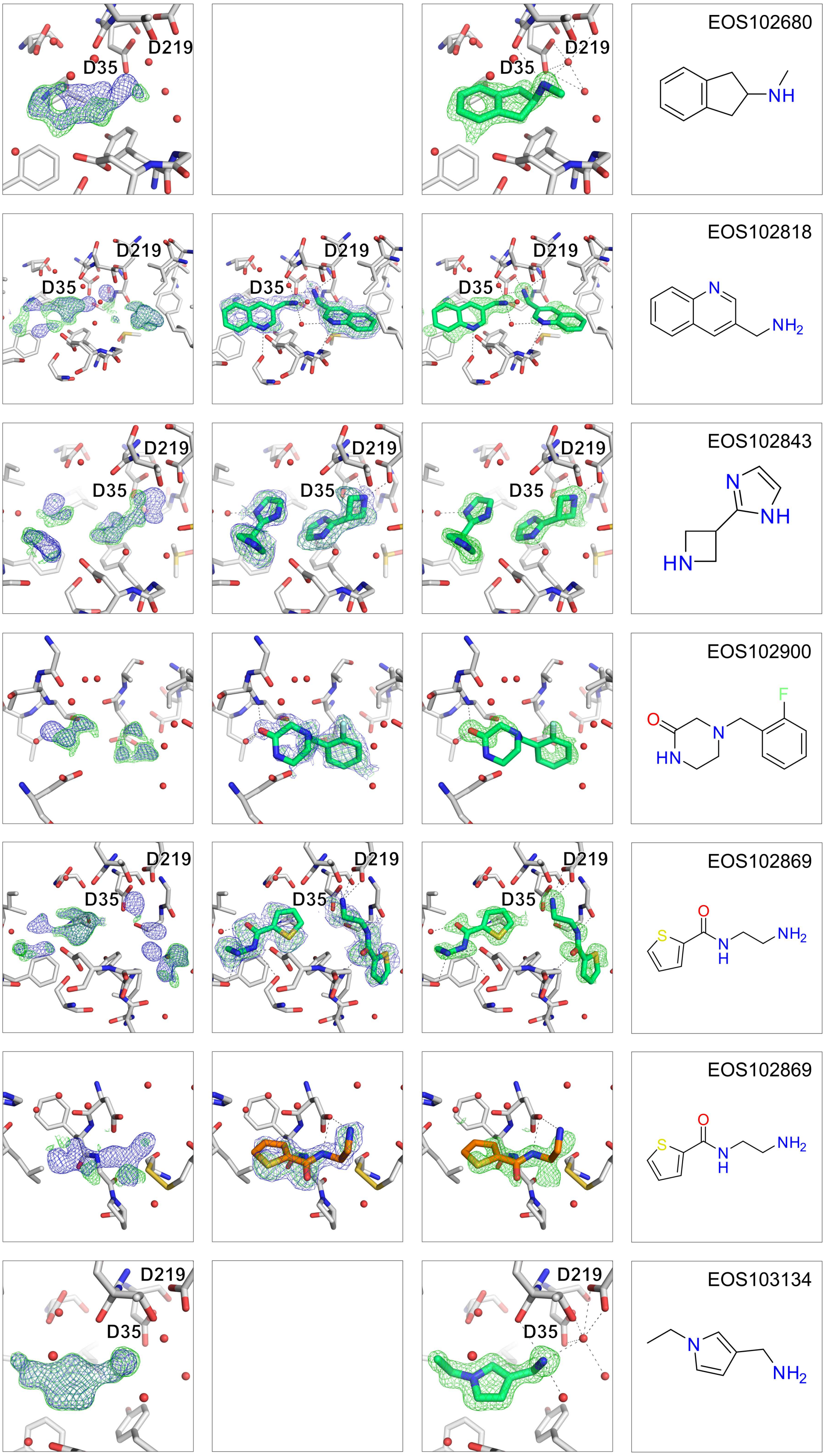
D35
D219
EOS102680
EOS102818
EOS102843
EOS102900
EOS102869
EOS102869
EOS103134

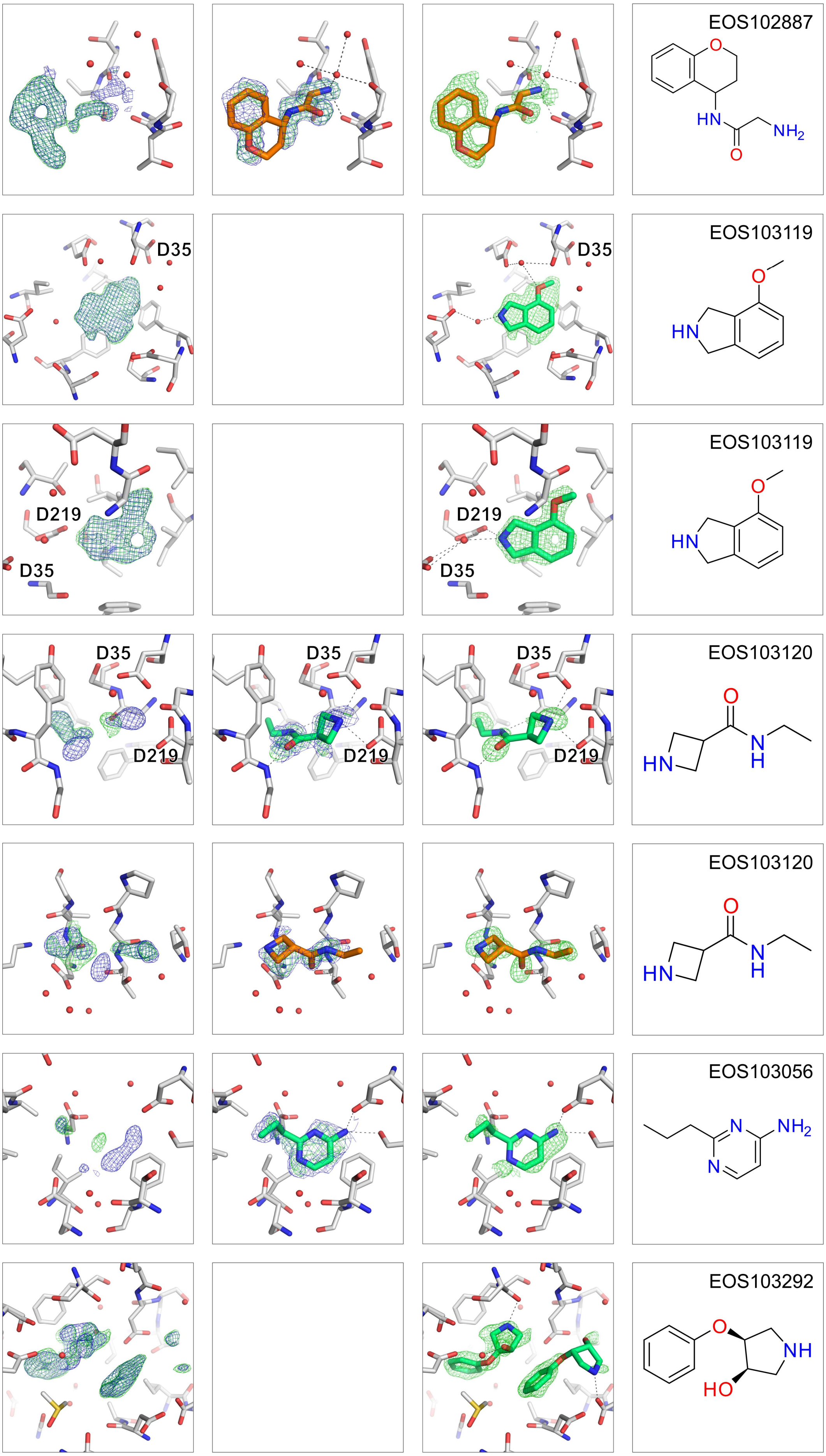
EOS102887
O
HN
O
NH2
D35
EOS103119
O
HN
D219
D35
EOS103119
O
HN
D35
D219
EOS103120
O
HN
N
H
EOS103120
O
HN
N
H
EOS103056
N
NH2
N
EOS103292
O
NH
HO

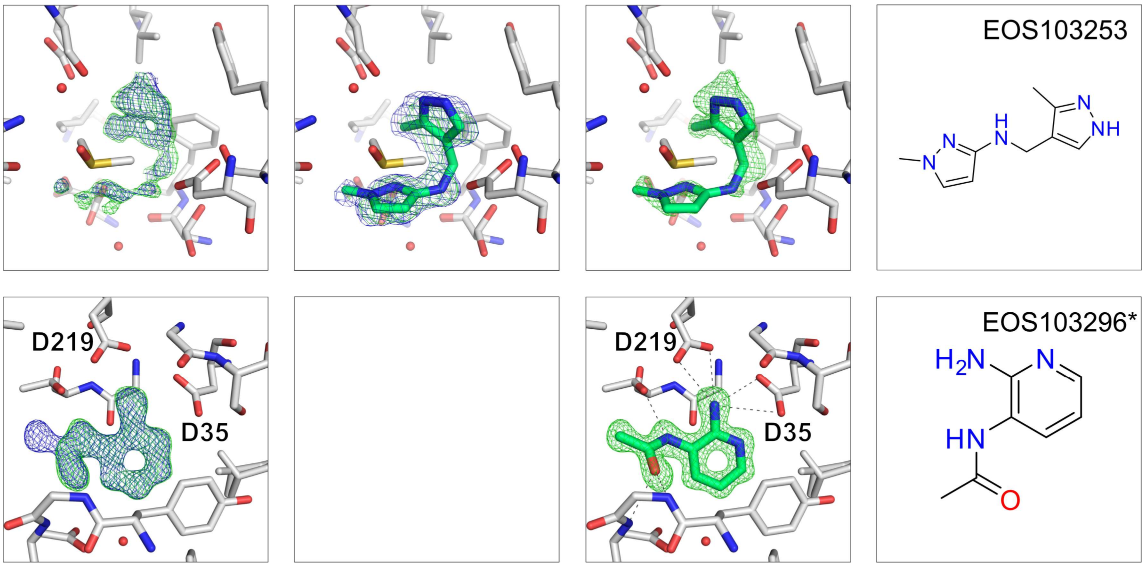

Supplemental figure 1. The additional fragment hits not used for follow-up search are depicted. The four panels from left to right show first the ($2mF_o - DF_c$, α_c) maps contoured at σ = 1 (blue) and the ($mF_o - DF_c$, α_c) maps contoured at σ = 3 (green) after auto-refinement with fspipeline, the second panel depicts for low occupancy fragments additionally the PanDDA event map shown at σ = 2 and PanDDA z-map shown at Z = 3 are shown, while for high occupancy binders no PanDDA maps are shown, the third panel depicts the omit-map around the ligand after refinement with the ligand placed and the fourth the chemical structure of the fragment hit. The catalytic residues Asp35 and Asp219 are indicated whenever the ligand is bound in proximity of the catalytic dyad. Peptide cleft binders are shown in green, remote binders in orange. Binder EOS103298* is not the actual compound EOS103296 but a precursor of compound EOS103296 still found in traces in the compound.

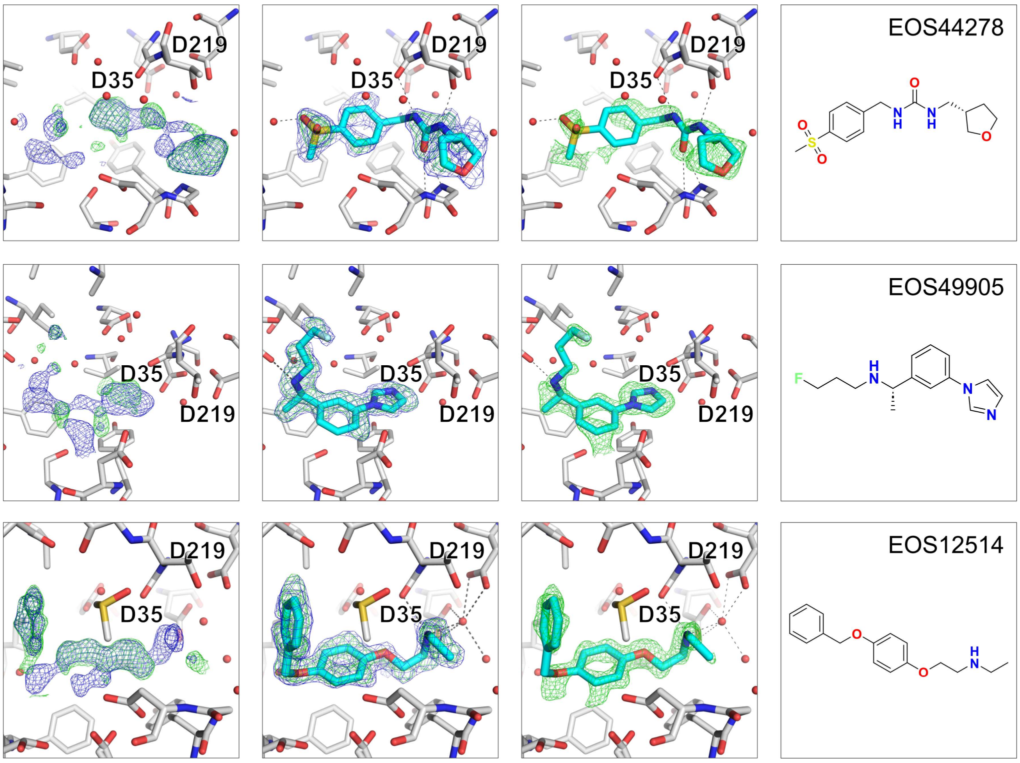

Supplemental Figure 2. Additional four follow-up binders of EP with lower occupancy than the initial fragment hits. The four panels from left to right show first the ($2mF_o - DF_c$, α_c) maps contoured at σ = 1 (blue) and the ($mF_o - DF_c$, α_c) maps contoured at σ = 3 (green) after auto-refinement with fspipeline, the second panel the PanDDA map with the event-map in blue contoured at σ = 2 and the z-map contoured at Z = 3 in green, next the omit-map around the ligand after refinement with the ligand placed in green and last the chemical structure of the fragment hit. The catalytic residues Asp35 and Asp219 are highlighted.

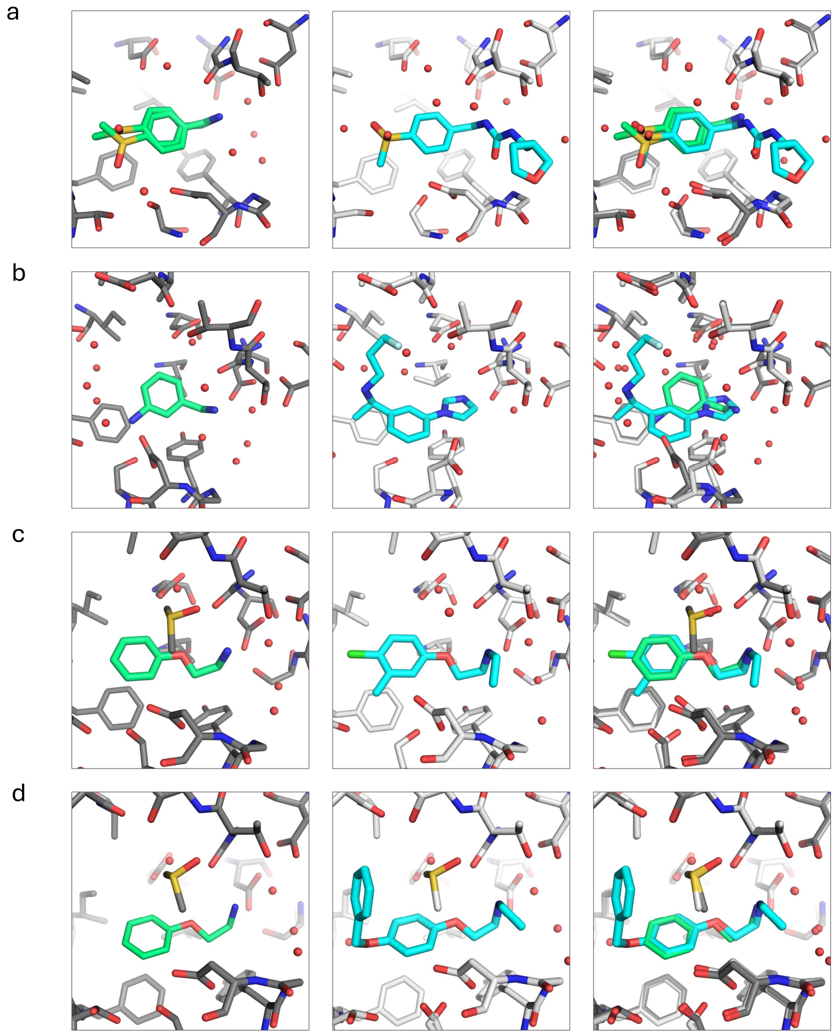

Supplemental figure 3. Comparison of initial fragment hit binding pose and follow-up compound binding pose of additional follow-up binders. The three panels from left to right show first in green the initial fragment hit, in the middle the follow-up compound in cyan and in the last panel an overlay of fragment hit and follow-up compound. Shown are from top to bottom EOS44278 (a), EOS49905 (b), EOS12482 (c) and EOS12514 (d).

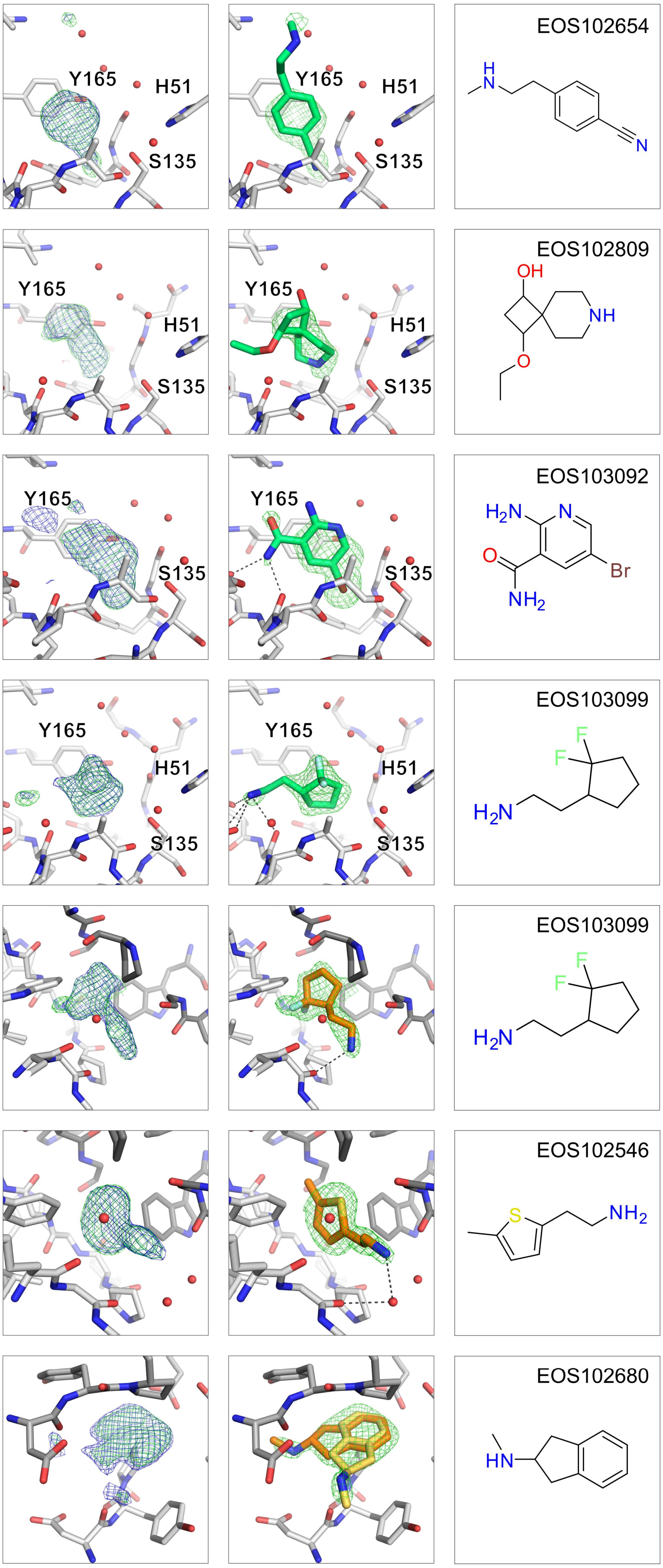
Y165
H51
S135
EOS102654
N
H
N
EOS102809
OH
NH
O
EOS103092
H2N
N
O
NH2
Br
EOS103099
F
F
H2N
EOS103099
F
F
H2N
EOS102546
S
NH2
EOS102680
HN

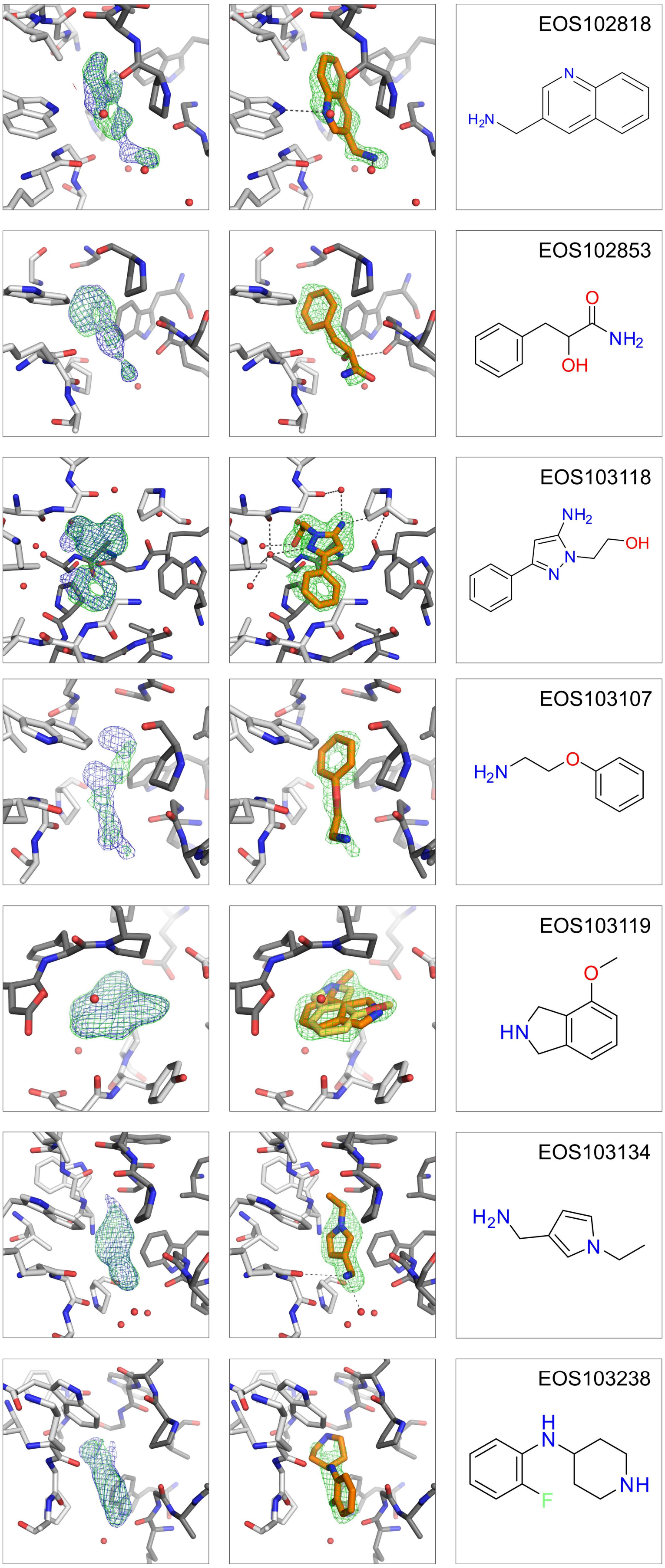
EOS102818
N
H2N
EOS102853
O
NH2
OH
EOS103118
NH2
OH
N
N
EOS103107
H2N
O
EOS103119
O
HN
EOS103134
H2N
N
EOS103238
H
N
F
NH

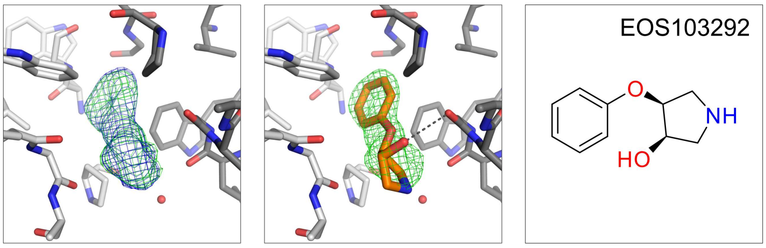

Supplemental figure 4. The additional fragment hits not used for follow-up search are depicted. The three panels from left to right show first the ($2mF_o - DF_c$, α_c) maps contoured at $\sigma = 1$ (blue) and the ($mF_o - DF_c$, α_c) maps contoured at $\sigma = 3$ (green) after auto-refinement with fspipeline, the second panel depicts the omit-map around the ligand after refinement with the ligand placed and the third panel the chemical structure of the fragment hit.

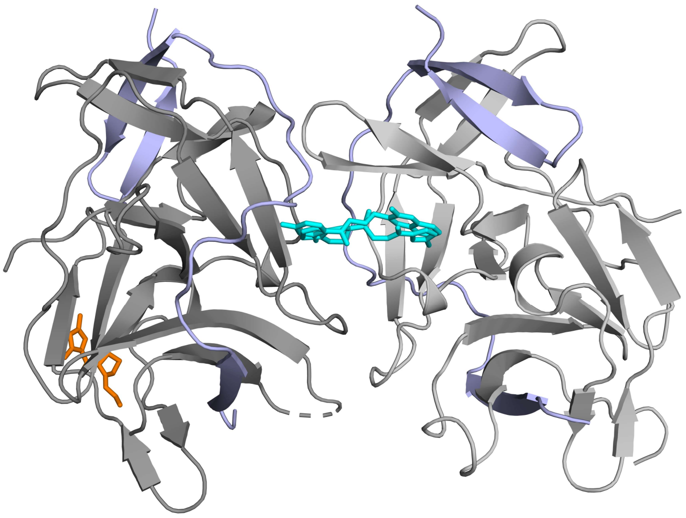

Supplemental Figure 5. Overview of all three NS2B-NS3 Zika protease binding events and their binding site. Binders close to the active site are shown in cyan, the remote binder in orange. The NS2B co-factor is colored in chain A and C in light blue, the NS3 protease domain is colored in dark grey in chain B and in light grey in chain D.

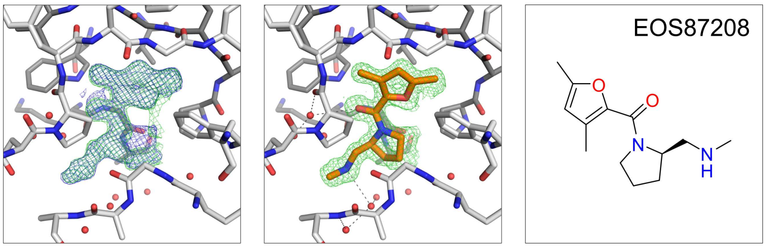

Supplemental Figure 6. NS2B-NS3 Zika protease remote follow-up hit with detailed presentation of follow-up hit. The three panels show from left to right the electron density map after auto-refinement with fspipeline, the ($2mF_o - DF_c$, α_c) maps are contoured at $\sigma = 1$ (blue) and the ($mF_o - DF_c$, α_c) maps contoured at $\sigma = 3$ (green), next the ligand modelled into the omit map after refinement and last the chemical structure of the binder.
